# Changes in ion transport in inflammatory disease

**DOI:** 10.1186/1476-9255-3-5

**Published:** 2006-03-29

**Authors:** Michael Eisenhut

**Affiliations:** 1 Institute of Child Health, University of Liverpool, Eaton Road, Liverpool, L12 2AP, UK

## Abstract

Ion transport is essential for maintenance of transmembranous and transcellular electric potential, fluid transport and cellular volume. Disturbance of ion transport has been associated with cellular dysfunction, intra and extracellular edema and abnormalities of epithelial surface liquid volume. There is increasing evidence that conditions characterized by an intense local or systemic inflammatory response are associated with abnormal ion transport. This abnormal ion transport has been involved in the pathogenesis of conditions like hypovolemia due to fluid losses, hyponatremia and hypokalemia in diarrhoeal diseases, electrolyte abnormalites in pyelonephritis of early infancy, septicemia induced pulmonary edema, and in hypersecretion and edema induced by inflammatory reactions of the mucosa of the upper respiratory tract. Components of membranous ion transport systems, which have been shown to undergo a change in function during an inflammatory response include the sodium potassium ATPase, the epithelial sodium channel, the Cystic Fibrosis Transmembrane Conductance Regulator and calcium activated chloride channels and the sodium potassium chloride co-transporter. Inflammatory mediators, which influence ion transport are tumor necrosis factor, gamma interferon, interleukins, transforming growth factor, leukotrienes and bradykinin. They trigger the release of specific messengers like prostaglandins, nitric oxide and histamine which alter ion transport system function through specific receptors, intracellular second messengers and protein kinases. This review summarizes data on in vivo measurements of changes in ion transport in acute inflammatory conditions and in vitro studies, which have explored the underlying mechanisms. Potential interventions directed at a correction of the observed abnormalities are discussed.

## Background

### Physiology of ion transport

**Figure 1:**
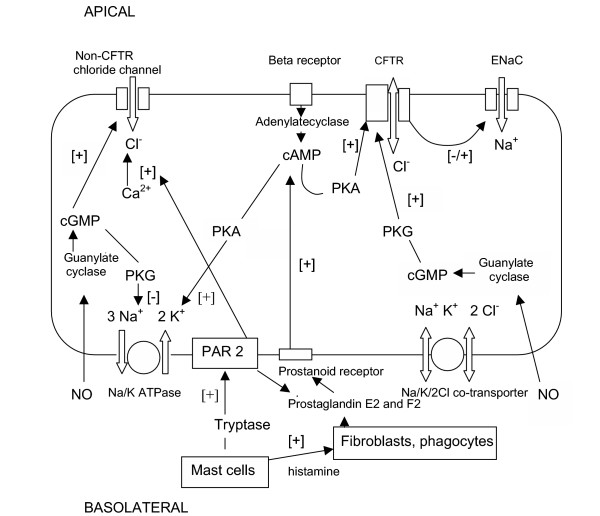
Components of epithelial ion transport and their interactions [[+] = activating, [-]= inactivating].

Ion transport across cell membranes and epithelial cell layers is the basis for generation of membrane potentials and provides the osmotic gradients for transmembranous and paracellular fluid transport. Transport of sodium [Na^+^], potassium [K^+^] and chloride [Cl^-^] are involved in maintenance of the membrane potential and Na^+ ^and Cl^- ^in fluid transport through creation of osmotic gradients. The main mechanisms for ion transport in the single cell as well as through epithelial cell layers involve for sodium the epithelial sodium channel [ENaC], which allows passive influx of Na^+ ^into the cell through the apical membrane and the sodium potassium ATPase [Na/K ATPase], which moves Na^+ ^actively out and K^+ ^into the cell in blood cells and the basolateral membrane of epithelial cells [See figure 1]. Water follows the so generated osmotic gradient probably paracellular and through aquaporine water channels in alveolar epithelial cells, renal tubular cells and colonocytes. Chloride transport in the apical epithelial cell membrane is mainly mediated by the cAMP dependent Cystic Fibrosis Transmembrane Conductance Regulator [CFTR] chloride channel, which can move chloride into and out of the cell, and the calcium activated chloride channels. Calcium activated chloride channels (CaCCs) complement the function of CFTR in transepithelial chloride and fluid transport [1,2]. Their molecular nature is uncertain. CFTR and CaCC seem to regulate each others activity: CFTR activation or increased expression is associated with a reduction in CaCC function. Regarding its role in transepithelial chloride and fluid transport most is known from experiments with airway epithelial cells. While CFTR regulates basal airway surface liquid [ASL] homeostasis, CaCCs seem to regulate Cl^- ^secretion and ASL height acutely and in response to extracellular stimuli [3].

The Na^+^/K^+^- 2Cl^-^- symporter channel permits co-transport of sodium and potassium and chloride through the baso lateral cell membrane in both directions. Other less important ion transport mechanisms include other apical Na-coupled transporters, such as Na^+^/phosphate, Na^+^/proton and Na^+^/amino acid antiports and Na^+^/glucose antiport systems which account for a small fraction of transepithelial sodium and liquid transport. The aims of this review are to summarize and interpret the current knowledge on influences of inflammation and its mediators on cellular ion transport in an attempt to identify common mechanisms of this interaction across all organ systems in humans. The review of the current knowledge may facilitate further research by stimulation of thinking across the boundaries of ion transport and inflammation research in separate medical subdisciplines. This is also an attempt to highlight pathways along which a correction of inflammation induced abnormalities of ion transport has been achieved by pharmacological intervention in previous studies.

## Methods

### Inclusion and exclusion criteria

The results of investigations of pathophysiological processes in humans involving inflammation-associated changes in ion-transport will be summarized. In vitro studies were only quoted if the investigated process offered a potential explanation of the processes observed in humans. Direct effects of microbial pathogens independent of an inflammatory process were excluded from the review. The keywords used for literature search were "ion transport", which was combined with "inflammation" and the terms "sodium channel", "chloride channel", "potassium channel", "CFTR" and "ATPase" combined with the terms "cytokine", "chemokine", "prostaglandin", "leukotriene" and "kinine". Databases searched were Pubmed, Medline [1951 to present] and EMBASE [1975 to present]. The reference lists of relevant articles were also scanned.

### Excluded studies

Reports on changes of Na/K ATPase function in skeletal muscle in endotoxemia in human and animal experiments [4] were excluded as a direct effect of the endotoxin rather than inflammatory mediators was involved. Studies of the effects on inflammatory mediators on membrane ion transport in myocardium are limited to animal and in vitro studies [5] and the significance of the findings to humans is unknown. The studies relating to this were therefore not included. Not included in this review were the in vitro findings regarding changes in ion transport in the middle ear epithelium [6] as there are no in vivo data on humans available supporting a pathophysiological role of changes in ion transport in middle ear disease. There were no studies on the interaction of inflammatory processes and ion transport in pancreas, salivary and sweat gland and epithelium of the genital tract. There were no relevant studies on changes of potassium channel function by inflammatory mediators.

### Explanation of methods used to measure ion transport

#### Nasal potential difference measurement

Nasal potential difference measurements involve the in vivo measurement of a potential difference [voltage] between a cutaneous or intravenous electrode and a fluid perfused electrode in contact to the respiratory epithelium under the inferior turbinate of the nose. The potential measured represents the end result of ion transport processes, which are dominated by sodium transport. On change of the perfusion fluid to an amiloride [ENaC blocker] containing fluid the resulting depolarization is equivalent to the contribution of ENaC function to the potential. A perfusion with a low chloride or chloride free solution plus amiloride following this measurement will activate chloride channels in a normal respiratory epithelium and result in a hyperpolarisation which represents the ability of the epithelium to secrete chloride and is thus a measure of chloride channel function.

#### Short circuit measurement with Ussing chamber

In the Ussing chamber an epithelium is inserted between two fluid filled chamber halves, which contain electrodes for stimulation and measurement of current and voltage. In the short circuit current [I_sc_] measurement the charge flow per time through the epithelium [between the two chamber halves] when the epithelium is short-circuited, i.e. the voltage clamped to 0 mV, is measured. Ion channel function is analyzed by measurement of current or voltage changes in response to blockers and stimulators of ion channel function. Amiloride is used to block sodium channels and forskolin, a stimulator of the adenylate cyclase, which activates CFTR mediated chloride secretion through increase in intracellular cAMP, is used to test CFTR function.

#### Patch clamp electrode measurements

A synonym of this term is continuous single electrode clamp. A smooth surfaced tip of > 1 micron diameter of a hollow electrode is pressed against a cell membrane and suction is applied inside the electrode. This pulls the cell membrane inside the electrode forming a tight seal. With the attached electrode currents through ion channels of the patch can be measured. By taking the patch of membrane out of the cell an "inside out patch" is formed, which enables the inside of the ion channels to be studied. An "outside out patch" is created by pulling a membrane ball with the patch out of the cell to study the properties of ion channels protected from the outside environment and not exposed to the cell content. "Inside out" and "outside out" are excised patch modes for single channel recording. Another variation of the method is the "perforated patch" by which a recording from the inside of the cell is taken. In perforated patch recording the membrane is perforated using fungicides like nystatin. The resulting situation is electrically similar to whole-cell recording but prevents washout of cytosolic factors.

#### ^125^I efflux assay

This assay is used to measure the activity of the CFTR chloride channel in vitro.

After incubation of cells with the chloride analogue ^125^I, which leads to its uptake into the cells, the gamma radiation in efflux fluid elicited by secretagogues like forskolin [see above] or ionomycin [a calcium ionophore used to stimulate calcium activated chloride channels] is measured. The radioactivity in the cells at a defined point in time is calculated as the sum of radioactivities released in subsequent efflux samples and the radioactivity in the cells at the end of the experiment. The studies are then conducted with the inhibitor of the outwardly rectifying chloride channel [ORCC] DIDS (4,4-diisothiocyano-dihydro stilbene-2-2'-disulfonic acid) and the inhibitor of both CFTR and the ORCC DPC (diphenylamine-2-carboxylic acid or N-phenylanthranilic acid). From the difference in activity is then the activity of CFTR calculated.

#### Rubidium 86+ flux studies

Rubidium flux studies measure the activity of the Na/K ATPase. This is achieved in vitro by measuring the Na/K ATPase mediated uptake of a potassium analogue, the gamma radiation emitting isotope Rubidium 86^+ ^into cells over time. The uptake study involves interruption of the uptake at defined time points after which the radioactivity of the cell population is measured with a gamma counter. The Na/K ATPase mediated uptake is measured by conducting the measurements with and without addition of ouabain, a specific inhibitor of the Na/K ATPase.

## Ion transport in inflammatory diseases of the respiratory system

### Upper respiratory tract

In patients with pollen allergy the nasal potential difference, which is a representation of nasal mucosal sodium transport was reduced in the pollen season compared to controls. Amiloride sensitive nasal potential difference, which reflects nasal epithelial sodium channel function was found to be significantly decreased after allergen challenge [7]. This supported the hypothesis that epithelial sodium transport is reduced during allergic inflammation. The findings may explain the increased epithelial surface fluid volume in the upper respiratory tract leading to excessive rhinorrhea in allergic rhinitis. A study on humans with chronic sinusitis investigated the correlation of the dynamic visco-elasticity of maxillary sinus fluid and change in amiloride responsive transmucosal short-circuit current in the Ussing chamber [8]. A significant positive correlation between visco-elasticity and sodium channel channel function was found. This suggested that the concentration of epithelial surface liquid volume in the maxillary sinus in sinusitis is dependent on changes in transepithelial sodium transport. In vitro studies have also found a reduction in CFTR gene and protein expression in chronically inflamed nasal polyps associated with a reduced cAMP-dependent short ciruit current in the Ussing chamber. This effect was reversible by removal of transforming growth factor beta 1 (TGF-beta 1) from the culture medium and could be reproduced by the addition of TGF-beta 1 to normal nasal mucosa [9].

### Lower respiratory tract

**Figure 2:**
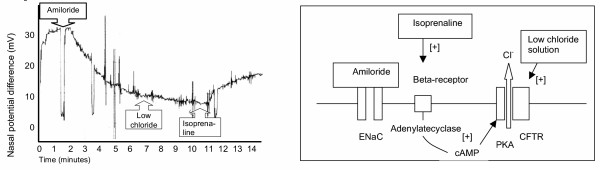
Nasal potential difference in a child ventilated with meningococcal septicaemia related pulmonary edema. The graph shows a lack of response of chloride channels in airway epithelium to a low chloride solution. This response is restored by the addition of the beta receptor agonist isoprenaline to the perfusate [unpublished graph from work published in [11]]. A model of the components of the ion transport systems involved has been put next to it: [+] = activating.

In vivo studies of ion transport in humans with systemic inflammatory response syndrome and pulmonary edema found evidence of a systemic reduction in epithelial sodium and chloride transport [10,11]. The studies investigated meningococcal septicemia induced pulmonary edema. Chloride channel function in patients with pulmonary edema was reduced on nasal PD measurements [See figure 2]. The extent of a reduction of systemic sodium and chloride transport as reflected in sweat and salivary sodium and chloride levels correlated significantly with the severity of respiratory compromise. The results were compatible with an inhibition of epithelial chloride transport through the CFTR and a sodium potassium ATPase dysfunction in children with meningococcal septicemia induced pulmonary edema. Hormonal regulators of the Na/K ATPase like aldosterone, cortisol and thyroxine were not decreased in these patients.

#### Regulation of ion transport protein activity

The CFTR dysfunction may have been related to the Na/K ATPase dysfunction and previous studies have demonstrated a reduction of function of the Na/K ATPase in red blood cells, muscle and liver of patients with septicemia [12,13]. Inhibition of the Na/K ATPase in human pulmonary Calu-3 cells lead to a reduced transcription of CFTR [14]. CFTR dysfunction was also induced through Na/K ATPase inhibition by ouabain in airway epithelial cells in another experiment [15]. CFTR dysfunction has been linked to reduced distal airways fluid clearance and fluid transport across alveolar epithelial type II cells [16,17]. There is evidence that in alveolar epithelial cells as opposed to upper airways epithelia a reduction in CFTR function is linked to a reduction in sodium transport through ENaC, which may contribute to the reduced lung liquid clearance observed in pulmonary edema. CFTR activation by cAMP in alveolar epithelial cells has been shown to be a condition for apical sodium influx into alveolar epithelial cells through ENaC by hyperpolarisation [18]. The lack of absorption of sodium associated with chloride channel dysfunction could explain a reduced alveolar liquid clearance because of the reduced osmotic effect drawing water through the alveolar aquaporin channels [19]. In isolated congenital CFTR dysfunction (cystic fibrosis) an increase in sodium transport by an increase in apical ENaC and basolateral Na/K ATPase activity in the airways can compensate for a reduction in alveolar chloride and sodium transport. In septicemia associated pulmonary edema this compensation is prevented by additional inhibition of the Na/K ATPase. Patients with septicemia generate high levels of pro-inflammatory cytokines like tumor necrosis factor [TNF] [20]. Studies in animal models of pulmonary edema found that tumor necrosis factor has a dichotomal role in its activity on sodium transport and pulmonary edema reabsorption [21]. Some studies found that incubation with TNF was associated with a reduction in alveolar epithelial cell sodium transport in vitro while others found that in an in vivo model of bacterial pneumonia in rats TNF increased alveolar fluid clearance. The enhancing effect of pneumonia with its high pulmonary TNF levels on alveolar fluid clearance was reduced by blockage of ENaC by amiloride [22]. This contradiction was recently resolved in a study in a rat model, in which TNF, when complexed with the soluble TNF receptor 1 construct [sTNFR1], increased alveolar fluid clearance via a lectin binding structure but without sTNFR1 it inhibited liquid clearance. STNFR1 seemed to modulate TNF function by diverting it from classical TNF receptors to an alternative receptor activating ENaC [21].

IL-1beta was found to antagonize the effects of prostaglandin E2 [PGE2] which stimulated chloride transport in canine tracheal epithelium and in Calu-3 bronchial epithelial cells by inducing excess production of PGE2. This lead to a down regulation of EP_4 _prostanoid receptors and subsequently to a reduction of PGE2 induced cAMP production. Reduced intracellular cAMP levels then lead to a reduction in cAMP dependent CFTR function [See figure 1] [23]. IL-1beta was also found to reduce ENaC function in human bronchial epithelial cells without reducing ENaC expression [24]. Another inflammatory mediator associated with reduced alveolar sodium transport and fluid clearance is nitric oxide [NO]. It is active by an unknown mechanism, which may involve an inhibition of the adenylate cyclase. This may, through inactivation of the Na/K ATPase, lead to a reduction of alveolar epithelial fluid transport [25]. NO is released by distal lung epithelial cells and alveolar macrophages in response to proinflammatory cytokines like IL- 1beta, TNF and interferon gamma [IFN gamma] [26-29].

More recently it has been recognized that the lipoxygenase product leukotriene D4 increases alveolar epithelial sodium transport and fluid clearance by activation of the lung Na/K ATPase [30].

Previous investigations into the influence of inflammatory mediators on respiratory epithelial chloride transport in vitro have demonstrated that PGE2 released in response to bradykinin [31], eosinophil major basic protein [32] and leukotrienes C4 and D4 [33] appeared to increase chloride secretion in the canine tracheal epithelium using short-circuit current measurments in the Ussing chamber. Leukotriene D4 seemed to be most effective. NO has been shown to activate non-CFTR chloride currents [perforated patch clamp technique] via a cyclic guanosine monophosphate [cGMP] dependent mechanism [34]. Other inflammatory mediators increasing chloride secretion have been reviewed [35] and include vasoactive intestinal peptide, which is the most abundant peptide in the human lung [36], substance P [37-39] and the neurokinins A and B [40]. The exact mechanism and channels involved are however unknown.

A new pathway for prostaglandin E2 release and stimulation of respiratory epithelial chloride secretion has been revealed recently: Activated mast cells can by secretion of tryptase stimulate protease activated receptors type 2 [PAR2] in airway epithelial cells [41]. PAR2 are induced by TNF and IL-1 [42]. Stimulation of these receptors lead directly and mediated through PGE2 release to an activation of calcium activated chloride channels in mouse and human airway epithelial cells [43].

#### Regulation of transport protein expression

Prolonged exposure of alveolar epithelial cells to TNF alpha by in vitro incubation [24hours], a condition which is known to change the physiological properties of alveolar epithelial cells significantly [19] seemed to reduce ENaC mRNA expression without impact on Na/K ATPase expression [44]. Similarly both IL-1beta and TGF- beta1 seemed to reduce alveolar epithelial sodium uptake in alveolar type II cells by reduction of expression of the alpha subunit of ENaC [45,46]. In the preterm fetal guinea pig lung however IL-1 beta increased both ENaC and Na/K ATPase expression [47]. Another group reported a TGF- beta1 induced increase in ENaC function as measured by short circuit current in rat alveolar epithelial cell monolayers without increase in expression of ENaC but an increase in expression of Na/K ATPase [48].

A reduction of beta and gamma units but not the alpha unit of ENaC was found to be an effect of IL-4 in vitro and this was associated with inhibition of the amiloride-sensitive Na^+ ^channel as measured in short circuit current experiments [49]. This cytokine is also a potent upregulator of CFTR protein expression and function in vitro [49,50]. Another cytokine, which was found to increase CFTR expression in human bronchial epithelial cells is IL-1 beta and this was associated with an increase in short circuit current in the Ussing chambers [24]. Antagonistic effects may have IFN gamma because short-circuit current measurements in the Ussing chamber of bronchial epithelial cells showed that it decreases CFTR dependent chloride secretion significantly, which was associated with a reduction in CFTR mRNA [49]. IFN gamma has on the other hand been found to activate non CFTR- chloride channel activity by stabilization of mRNA transcripts in human Calu-3 lung epithelial cells with an associated increase in short-circuit current in the Ussing chamber [51]. Expression of CaCC was found to be increased in response the Th2 cytokines IL-4, 9 and 13 in human primary lung culture systems [52].

## Ion transport in gastrointestinal inflammatory disease

Infective and noninfective causes of diarrhoea have been associated with fluid and electrolyte losses leading to dehydration, hypovolemia, hyponatremia and hypokalemia [53,54]. This is indicative of an impaired gastrointestinal epithelial ion transport in these conditions.

While secretory diarrhoea is caused by a direct effect of toxins on epithelial ion transport, mainly via cAMP mediated activation of luminal CFTR and the associated inactivation of ENaC, studies indicated that intestinal ion transport in inflammatory diarrhoea is changed by effects of inflammatory mediators on CFTR, ENaC and Na/K ATPase. The effects of these inflammatory mediators are transmitted through more complex processes involving membrane receptors, second messengers and changes in mRNA transcription.

### Regulation of ion transport protein activity

A common pathway for a change in ion transport in both infective [caused by bacteria like *Yersinia sp., E. coli *and *Listeria*] and noninfective [ulcerative colitis and Crohn's disease] types of inflammatory diarrhoea has recently been clarified. All forms of inflammatory diarrhoea are associated with hypersecretion of chloride into the gut lumen by activation of CFTR at the apical membrane of colonocytes drawing water with it by osmotic effects. Activated CFTR can inhibit ENaC [55] and therefore sodium absorption in the gut and this may be the main mechanism of fluid loss in diarrhoea by reduction in sodium driven fluid absorption. Illustrated was the importance of sodium absorption by the result of investigations of T-cell activation induced diarrhoea in mice. The investigations demonstrated that TNF and not IFN gamma mediated an inactivation of the Na/K ATPase. This effect was independent of nitric oxide and was present in mice without functional CFTR [56].

In the human colonic T84 cell line Na/K ATPase was inhibited by IFN gamma and NO and this was associated with an increase in cell volume and intracellular sodium concentration [57]. These contradictory findings may indicate species differences in the response to cytokines or represent a response to changes in cellular phenotype following in vitro incubation. Inhibition of the Na/K ATPase by IFN gamma was also observed in murine small intestine preparations and the intestinal epithelial Caco-2 cell line [58,59]. These effects were not mediated by NO in the murine intestinal preparation [58].

The pro-inflammatory cytokine TNF caused in ex vivo human colonic mucosa with attached submucosa an increase in chloride and potassium secretion, which was found to be dependent on the effect of prostaglandin E2 [60]. The PGE2 mediated effect of TNF and also of IL-1 beta and IL-3 in increase of intestinal chloride secretion was confirmed in studies in which cryptosporidium infected pig ileum and untreated chicken intestine was found to respond with increased short circuit current to these cytokines. The increase in short circuit current in these experiments was blocked by cyclooxygenase inhibitors.

The investigators found that colonic intestinal cell monolayers [T84 cell line] required comounting with TNF stimulated jejunal fibroblasts to generate an increased short circuit current, which was sensitive to PGE2 inhibition. This indicated that fibroblasts may also be involved in changes in epithelial ion transport in inflammatory bowel disease [61,62]. PGE2 was also involved in the effects of the proinflammatory neuropeptide substance P in an increase in chloride secretion measured in an Ussing chamber experiment in the rabbit colonic mucosa. The effect of substance P was hereby dependent on mast cell activity and histamine [63]. Another pathway of induction of PGE2 release in the gut is through NO and leukotriene D4, which both stimulated prostaglandin release [64,65]. PGE2 activated chloride secretion via its effect on vacuolated columnar colonic epithelial cells [66]. This effect was mediated through cAMP activated protein kinase A (PKA), which activated CFTR. Amongst all prostaglandins only PGE2 and PGF2 were found to stimulate chloride secretion in rabbit distal colonic mucosa via cAMP in this context [67].

In the gut NO increased intracellular cGMP levels, a second messenger, which induced Cl ^-^secretion by stimulation of CFTR through cGMP-regulated protein kinase G type II [68]. Nitric oxide donors were able to stimulate chloride secretion in human colonic mucosa [69]. Another mediator, which increased chloride secretion in T84 human intestinal cell monolayers, is the neutrophil derived 5'adenosine monophosphate which after conversion to adenosine acted on adenosine receptors on the apical membrane [70,71].

Cytokines which enhanced cAMP mediated chloride secretion in human small intestinal ex vivo preparations were IL-2 and IL-15 [72].

Recently a new mechanism for stimulation of chloride secretion has been investigated. It involved the induction and activation of the proteinase activated receptor type 2 in epithelial cells by intestinal mast cells leading to activation of chloride channels by the same mechanism as found in the respiratory tract [41] [see above]. The essential role of mast cells in changes of ion transport in response to allergic inflammation has been confirmed by a study in which jejunal mucosa from rats responded with an increase in short-circuit current to antigen, a response which was inhibited by a chloride channel blocker and mast cell stabilizers [73]. Another group reported that the activation of chloride transport as measured by short circuit measurement in allergic inflammation in guinea pig colon was dependent on a pathway containing both histamine and prostaglandin [74].

IL-4 and IL-10 seem to counteract effects of pro-inflammatory mediators on chloride secretion in the gut. They have been found to diminish Cl^–^secretion, as measured by epithelial short ciruit current in the Ussing chamber [75-77]. IL-10 reduced chloride secretion by reduction of intracellular cAMP and mediated increased sodium and chloride absorption as measured under voltage clamp conditions in the Ussing chamber in rat small intestine [76]. IFN gamma was also found to inhibit chloride secretion [58,78]. Sodium and chloride secretion was increased by addition of histamine, which acted via H1-receptors in porcine distal colon epithelial cells in the Ussing chamber [79]. TNF was found to potentiate histamine induced ion secretion in the HT29cl.19A cells and mouse distal colon [80]. However the response of the short circuit current to mast cell mediators was found to be decreased in human small and large bowel mucosa-submucosa preparations treated with histamine suggesting interspecies differences [81].

### Regulation of transport protein expression

Experiments with the gut derived epithelial cell lines T84 and HT-29 showed that interferon gamma and TNF both independently and if applied together synergistically, reduce CFTR mRNA by destabilization of transcripts and with prolonged [> 24 hour] exposure also CFTR protein on the cell surface. This process seemed to reduce the chloride current [82]. Other cytokines which have been found to reduce CFTR mRNA and intracellular CFTR protein expression and integration in the apical membrane leading to a reduced epithelial secretory response in colonic epithelia [T84 and HT-29] were TGF beta 1 and interleukin-4 [83]. Other studies have demonstrated a NO induced reduction in chloride secretion, which appears to be related to an inhibition of cAMP dependent CFTR trafficking inside intestinal epithelial cells [84]. Colitis in rats was associated with a long term increase in inducible NO-synthetase expression leading to persistently reduced chloride secretion in response to stimuli [85].

**Figure 3:**
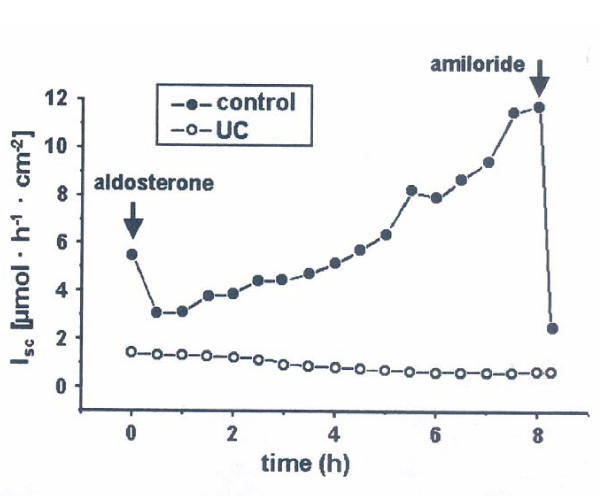
Short-circuit measurements on healthy colonic mucosa and mucosa of a patient with ulcerative colitis [UC]. Shown is the response of the short circuit current (I_SC_) to aldosterone [upregulator of ENaC and Na/K ATPase function] and amiloride [ENaC blocker] [Taken with permission from [89].

A dichotomous effect on CFTR expression was noted for Interleukin 1beta. It upregulated CFTR mRNA levels at a dose of 0.25 ng/ml and inhibited CFTR mRNA and protein expression at higher doses [1 ng/ml] in T 84 cells [86]. This cytokine increased protein expression of the Na K 2Cl symporter and reduced Na/K ATPase protein expression in another experiment [87]. TNF was found to have the opposite effect on Na K 2Cl symporter expression in rat colon where it reduced both Na/K ATPase and Na K 2Cl symporter expression and activity in surface and crypt colonocytes [88]. This effect was dependent on PGE2 and inhibitable by indomethacin. In an ex vivo human colonic mucosa plus submucosa preparation of patients with ulcerative colitis aldosterone was unable to stimulate electrogenic sodium transport in the Ussing chamber or ENaC mRNA levels and protein expression [See figure 3]. This lack of response to aldosterone could be reproduced by incubation of healthy human colonic tissue with TNF and IFN gamma, which reduced electrogenic sodium transport and lead to a reduced upregulation of ENaC mRNA [89]. Another group reported that the expression of beta-and gamma-subunits of ENaC was inhibited by both TNF and IL-1 beta in rat distal colon. IFN gamma had no effect on ENaC expression in this study [90].

IFN gamma reduced Na/K/2Cl-cotransporter and Na/K ATPase expression without altering CFTR levels in T84 intestinal epithelial cells [91]. Another study however noted that interferon gamma reduced Na/K ATPase activity without change in Na K ATPase expression in human intestinal Caco-2 cells [59].

This may explain the dysfunction of the apical Na^+ ^-glucose cotransport found in the chronically inflamed rabbit ileum for which these basolateral pumps are essential for creating a sodium gradient [92]. Interleukin-4 in contrast to the genetically closely linked interleukin -13 was able to reduce chloride secretion as assessed by ^125 ^I efflux assay by down regulation of CFTR expression in the human intestinal cell line T84 in vitro [93].

## Ion transport in pyelonephritis

In a retrospective study of 300 children with urinary tract infection 6.8% were noted to have significant hyponatremia and hyperkalemia [94]. This phenomenon has been investigated further and fractional renal potassium excretion and transtubular potassium concentration gradient were found to be reduced in children with pyelonephritis [95]. This condition, which resolved on recovery, was termed transient pseudohypoaldosteronism as aldosterone levels were found to be normal or elevated [96].

### Regulation of ion transport protein activity

The underlying mechanism is related to an impairment of renal sodium and potassium transport by inflammatory mediators. Interleukin-1 infusion was found to induce natriuresis in rats [97]. In vitro studies on inner medullary collecting duct cells showed that IL-1 was able to reduce the ouabain sensitive Rb 86^+ ^flux indicating a reduction in Na/K ATPase activity [98]. The same was found for an interaction of endothelial cells and the proximal tubular epithelial cells in their vicinity in a co-culture experiment where endothelial derived IL-1 and bradykinin, mediated by NO and cGMP, reduced sodium transport by down regulation of Na/K ATPase activity [99]. The effect of IL-1 was inhibited by a cyclooxygenase inhibitor [indomethacin], suggesting that the effect is mediated by prostaglandins [100]. PGE2 inhibited the Na/K ATPase in inner medullary collecting duct cells and cortical collecting duct in vitro [101,102]. PGE2 was also found to lead to cellular edema in isolated perfused rabbit collecting duct cells, an effect seen with the known Na/K ATPase inhibitor ouabain. This effect of PGE2 seemed to vary across different anatomical sections of the renal tubule: In the rat kidney medullary Na/K ATPase was only moderately suppressed while a more significant suppression was found in the thick ascending limb of the loop of Henle [103].

It has been suggested that PGE2 doesn't act directly on the Na/K ATPase but through action on apical Na^+ ^entry. Patch clamp experiments showed that PGE2 reduced the open probability of apical sodium channels in rabbit cortical collecting tubules. This effect involved the release of inositolphosphate-3-sensitive intracellular calcium stores and calcium dependent activation of the apical membrane protein kinase C [104].

More potent directly inhibitory effects on the Na/K ATPase seem to come from substances which increase intracellular cAMP. The most important examples are epoxides, which are arachidonic acid metabolites of the cytochrome P450-dependent monooxygenase pathway, like 11,12 -dihydroxyeicosatrienoic acid, 5,6-eoxyeicosatrienoic acid and 12 [R]-hydroxyeicosatetraenoic acid [105].

The effect of nitric oxide on ion transport in the nephron has been reviewed recently [106]. One group reported that in rat kidney medullary slices NO donors mediated by cGMP and proteinkinase G inhibited Na/K ATPase activity [107]. This suggested that cytokine induced NO may be involved in changes in renal sodium and potassium transport.

In vivo studies have demonstrated that bradykinin is another potent natriuretic agent. This effect is mediated by inhibition of ENaC and Na/K ATPase activity possibly mediated by an increase in intracellular calcium and/or pH. Bradykinin seemed to counteract the stimulatory effect of Angiotensin [1-7] on the Na/K-ATPase activity of the basolateral membrane of the proximal tubule through a pathway involving phospholipase A2 and PGE2 [108]. Bradykinin revealed dichotomous effects in another study where it was found to increase Na/K ATPase activity through interaction with B1 bradykinin receptors but inhibited Na/K ATPase function by interaction with the B2 bradykinin receptors on renal proximal tubular cells in vitro [109].

### Regulation of transport protein expression

IL 1beta inhibited protein expression of Na/K ATPase in medullary and cortical rat kidney cells and this effect was mediated by the extracellular signal regulated protein kinase pathway which activated the nuclear factor NF-kappaB, thus leading to increased cycloxyenase-2 [COX-2] expression and PGE2 release. PGE2 in turn inhibited NF-kappaB and reduced the protein expression of Na/K ATPase [100]. Another explanation for a reduced Na/K ATPase activity in inflammatory renal disease provided the finding of a reduced binding of aldosterone to lymphocytes of patients with pyelonephritis [110]. A reduced aldosterone effect on tissues could lead to a reduction of Na/K ATPase expression and activity.

An increase in expression of apical amiloride-blockable Na+ channels was observed in a distal nephron cell line [A6] not after short [3–6min] but prolonged [10–50min] stimulation with PGE2. Short stimulation resulted in a reduced opening probability of sodium channels [111]. PGE2 also decreased the expression of the Na/K/2Cl cotransporter in mouse medullary thick ascending limb cells as measured by specific [3H] bumetanide binding [112].

Very little data are available for influence of cytokines on renal chloride transport. TGF beta1 was found to lead to perinuclear accumulation of CFTR protein and reduced chloride secretory responses to cAMP stimulating agents [82]. Bradykinin activated CaCCs in whole cell patch clamp experiments in mouse inner medullary collecting duct epithelium and this was associated with an increase in mRNA expression of this channel [113]. The basolateral PAR2 induced chloride secretion by CaCCs in M-1 mouse renal cortical collecting duct cells in short circuit experiments [114]

## Effects of changes in ion transport on inflammatory mediator production

Before considering interventions in the pathophysiological processes leading to changes in ion transport in inflammatory conditions, the physiological effect of a change in ion transporter function on inflammatory mediators needs to be considered. In vitro studies have investigated the effects of CFTR dysfunction in cystic fibrosis [CFTR mutation] patients on cytokine secretion in white blood cell and respiratory epithelial cell populations. Experiments revealed that blood mononuclear cells and lymphocytes with CFTR dysfunction exhibited a reduced potential for secretion of the cytokines IL-10 and IFN gamma [115,116]. The basal level of IL-8 secretion in monocytes from cystic fibrosis patients was significantly increased in vitro and the 50% effective concentrations for LPS-induced IL-8 production for both CF patients and obligate heterozygotes were 100-fold lower than in controls [117]. In a human pulmonary epithelial cell line bearing the Delta F508 mutation of CFTR resulting in a dysfunctional CFTR chloride channel, PGE2 secretion was significantly increased. This PGE2 release was attenuated by the experimentally induced retrafficking of the Delta F508-CFTR to the plasma membrane [118]. On the other hand the chloride channel blocker 5-nitro-2- [3-phenylpropylamino]-benzoic acid was able to inhibit PGE2 secretion in rat renal glomerular mesiangial cells in vitro [119]. Expression of epithelial inducible nitric oxide synthase and NO production was reduced in a human trachea epithelial cell line where CFTR activity was blocked by the over-expression of the CFTR regulatory domain. This was confirmed in CFTR deficient mice in vivo [120]. CFTR was moreover found to increase RANTES expression independently of its function as a chloride channel in bronchial cell lines [121,122].

These findings of changes in inflammatory mediator production related to CFTR dysfunction demonstrate that therapeutic modifications of ion transport abnormalities explained below may have an impact on the physiology of inflammatory processes.

## Interventions to modify ion transport

Interventions which modify ion transport in inflammatory disease can be directed at a reduction of production or activity of cytokines, their messengers like prostaglandins and nitric oxide and ion channels directly or through second messengers. Interference with the cytokine or chemokine network could interfer with their beneficial effects.

To overcome this a synthetic peptide [tip peptide] mimicking the lectine like domain of TNF has been constructed and was found to increase edema absorption [123]. Topical application of cytokines, hormones or hormone analogues, which directly or indirectly affect the function of ion transport systems can counteract the effects of inflammatory mediators on ion transport without weakening the systemic immune response. An example is epidermal growth factor [EGF], which has been shown to increase cAMP stimulated ENaC mediated sodium transport in the mouse model of colitis. The effect was achieved partly by increase of trafficking of ENaC containing intracellular vesicles to the apical membrane leading to increased insertion of the channel and partly by activation of ENaC through extracellular signal-regulated kinase, phosphatidylinositol 3-kinase and protein kinase C [124]. This can explain the reduction in diarrhoea in colitis, which was achieved by administration of EGF enemas in patients suffering from ulcerative colitis [125].

The beta-agonist isoprenaline has been shown to reverse chloride channel dysfunction in nasal respiratory epithelium in meningococcal septicemia related pulmonary edema in vivo [see figure 2]. Experiments with alveolar epithelial cells in vitro have demonstrated that CFTR in these cells can be activated by the beta agonists terbutaline [126], which is available as an aerosol for application in patients and licenced as asthma treatment. The mechanism for this effect has been investigated recently in the Calu-3 human bronchial epithelial cell line and involves stimulation of the adenylate cyclase by isoprenaline via beta-2 receptors [See ion transport model in figure 2]. The adenylate cyclase increases cAMP levels, which activate CFTR via proteinkinase A. IL-1beta has been found to enhance this isoprenaline induced cAMP accumulation through an up regulation of beta-2-receptors [23]. With regards to conditions with excessive CFTR function it is promising that nonsteroidal anti-inflammatory drugs including aspirin, ibuprofen and indomethacin reduced CFTR transcripts and subsequently cAMP-stimulated anion fluxes in T-84 colonic cell lines [127].

In the lung beta-agonists and dopamine have also been shown to improve sodium and the associated fluid transport in pulmonary oedema [128,129]. This is for beta agonists achieved by cAMP mediated activation of Na/K ATPase function and subsequently ENaC function.

Other hormones able to upregulate Na/K ATPase levels in cell membranes are glucocorticoids, insulin and thyroid hormones [130,131] but they have not been investigated for their effects on lung fluid clearance. Gene transfer of the beta1 subunit of the Na/K ATPase into rat lungs using electroporation increased lung liquid clearance significantly [132].

## Conclusion

### General principles of the interaction of inflammatory mediators with epithelial ion transport systems

Changes in ion transport were associated with inflammation due to allergy, auto-immune disease and infection. This means that not the etiological agents but mediators of inflammation, which are found in all types of inflammatory disease are involved in the changes in ion transport.

In respiratory tract, gut and kidneys pro-inflammatory cytokines are involved in down regulation of sodium transport. This down regulation affects not only protein function but also gene expression of ENaC and/or Na/K ATPase. This effect on function and gene expression is mediated by PGE2 and/or NO [See table 1]. PGE2 supports the inhibition of ENaC at the apical cell membrane by inducing a concomitant reduction in the activity or expression of the Na/K ATPase at the basolateral membrane. In all tissues the reduction in sodium transport was found to be refractory to normal or increased levels of aldosterone. Influences of inflammatory mediators on chloride transport have been detected in pulmonary, gut and kidney cells. Where investigated PGE2 seemed to simultaneously activate chloride transport and reduce sodium transport. The PAR2 receptor system seemed to be involved in regulation of chloride transport in all organ systems. Messengers involved in translating the signal of inflammatory mediators intracellularly are cAMP and cGMP leading both to protein kinase mediated modulation of CFTR-and Na/K ATPase function.

**Table 1:** Effects of inflammatory mediators on epithelial ion transport protein activity and expression.

**Type of ion transport** ** system **[references]	**Respiratory tract**								**Gut**									**Kidney**			
	TNF	IL-1	IL-4	IL-9	IL-13	IFNg	TGF	Leukotrienes	TNF	IL-1	IL-3	IL-4	IL-10	IFNg	TGF	Subst P	bradykinin	IL-1	TGF	bradykinin	epoxide
**CFTR**																					
activity [9,23,24,49,50,82,83,93]		+/- ^a,b^	+			-	-	+^a^	+ ^a,b^	+ ^a,b^	+ ^a^	-	-	-	-	+ ^a,c^	+		-		
expression [9, 24, 49, 50, 82, 83, 86, 93]		+	+			-	-		-	+/-		-		-	-				-		
**ENaC**																					
activity [21, 24, 45, 46, 48, 49]	+/- ^a,b^	-	-				+													-	
expression [44, 45, 46, 47, 49, 89, 90]	-	+/-	-				-		-	-				-							
**Na/K ATPase**																					
activity [30, 56, 57, 58, 59, 88, 98, 99, 108, 109]								+^a^	-					-	-			-^a,b^		+/-^a^	-
expression [47, 48, 87, 88, 91, 100]		+					+		-^a^	-				-				-			
**Non CFTR Cl**^-^**channel**																					
activity [51, 133]	+	+				+															+
expression [51, 52, 113]			+	+	+	+															+
**Na/K 2Cl**^-^**co-transporter**									-												
activity [88]									-												
expression [87, 88, 91]									-^a^	+				-							

+ = increase

-=decrease

+/- = increase or decrease depending on experimental conditions and possibly second messenger involved

^a ^mediated through PGE2

^b ^mediated through NO

^c ^mediated through histamine

### Directions for future research

This review reveals the need for more research into most aspects of the interaction of pro-and anti-inflammatory mediators and ion transport systems. Table 1 shows that many mediators have only been investigated for their impact on ion transport in one organ system and not in other systems where they may be equally or more important in regulation of sodium and chloride transport in health and disease. The ion channels targeted by inflammatory mediators like VIP, neurokinins A and B and eosinophil major basic protein have not been identified yet and analysis of the interactions involved requires further research. Contradictory findings in cytokine effects on epithelial ion transport, which have been resolved for TNF [regulation by TNF receptor], IL-1beta [dependency on concentration and presence of a beta agonist] and bradykinin [presence of B1 or B2 bradykinin receptor], need to be addressed for other mediators. TGF beta1 has been found to increase ENaC function in some but decrease ENaC function in other experiments on alveolar epithelial cells. NO seemed to increase chloride secretion in colonocytes in one study but reduced it in intestinal cells in another experiment. Interleukin-4 increased expression of CFTR in lung epithelial cells but decreased its expression in colonic epithelial cell lines. A detailed understanding of potential dichotomous effects of inflammatory mediators may, as illustrated above for TNF, lead to a deeper understanding of the pathophysiology of ion transport in inflammatory disorders and open avenues for new therapeutic approaches.

The relative importance of each of the numerous inflammatory mediators involved in regulation of ion transport needs to be clarified for each type of ion transport system and the various organsystem by comparative studies. This will facilitate the therapeutic targeting of the most important pathophysiological processes. Topical therapies in the colon and lungs may be able to deliver agents counteracting undesirable effects of inflammatory mediators on epithelial ion transport without systemic side effects. This may lead to supportive therapies directed specifically at a correction or prevention of the abnormal fluid and electrolyte shifts found in inflammatory diseases affecting these organ systems.

## Abbreviations

cAMP Cyclic adenosine monophosphate

cGMP Cyclic guanosine monophosphate

CaCC Calcium activated chloride channel

CFTR Cystic fibrosis transmembrane conductance regulator

COX-2 Cyclooxygenase-2

EGF Epithelial cell growth factor

ENaC Epithelial sodium channel

IFNg Interferon gamma

IL Interleukin

Na/K ATPase Sodium potassium adenosine triphosphatase

NO Nitric oxide

PAR2 proteinase activated receptor type 2

PGE2 Prostaglandin E2

PK Protein kinase

RANTES Regulated upon activation, normal T-cell expressed and secreted

Rb Rubidium

TGF Transforming growth factor

TNF Tumor necrosis factor alpha

TNFR Tumor necrosis factor receptor

## Competing interests

The author(s) declares that he has no competing interests.

## Authors' contributions

The author was the only person involved in the production of this review.
